# Arsenic-Redox Transformation and Plant Growth Promotion by Purple Nonsulfur Bacteria* Rhodopseudomonas palustris* CS2 and* Rhodopseudomonas faecalis* SS5

**DOI:** 10.1155/2017/6250327

**Published:** 2017-03-12

**Authors:** Kanza Batool, Fatima tuz Zahra, Yasir Rehman

**Affiliations:** Department of Microbiology & Molecular Genetics, University of the Punjab, Lahore, Pakistan

## Abstract

Arsenic (As) is a well-known toxic metalloid found naturally and released by different industries, especially in developing countries. Purple nonsulfur bacteria (PNSB) are known for wastewater treatment and plant growth promoting abilities. As-resistant PNSB were isolated from a fish pond. Based on As-resistance and plant growth promoting attributes, 2 isolates CS2 and SS5 were selected and identified as* Rhodopseudomonas palustris* and* Rhodopseudomonas faecalis*, respectively, through 16S rRNA gene sequencing. Maximum As(V) resistance shown by* R. faecalis* SS5 and* R. palustris* CS2 was up to 150 and 100 mM, respectively.* R*.* palustris* CS2 showed highest As(V) reduction up to 62.9% (6.29 ± 0.24 mM), while* R. faecalis* SS5 showed maximum As(III) oxidation up to 96% (4.8 ± 0.32 mM), respectively. Highest auxin production was observed by* R. palustris* CS2 and* R. faecalis* SS, up to 77.18 ± 3.7 and 76.67 ± 2.8 *μ*g mL^−1^, respectively. Effects of these PNSB were tested on the growth of* Vigna mungo* plants. A statistically significant increase in growth was observed in plants inoculated with isolates compared to uninoculated plants, both in presence and in absence of As.* R. palustris* CS2 treated plants showed 17% (28.1 ± 0.87 cm) increase in shoot length and 21.7% (7.07 ± 0.42 cm) increase in root length, whereas* R. faecalis* SS5 treated plants showed 12.8% (27.09 ± 0.81 cm) increase in shoot length and 18.8% (6.9 ± 0.34 cm) increase in root length as compared to the control plants. In presence of As,* R. palustris* CS2 increased shoot length up to 26.3% (21.0 ± 1.1 cm), while root length increased up to 31.3% (5.3 ± 0.4 cm), whereas* R. faecalis* SS5 inoculated plants showed 25% (20.7 ± 1.4 cm) increase in shoot length and 33.3% (5.4 ± 0.65 cm) increase in root length as compared to the control plants. Bacteria with such diverse abilities could be ideal for plant growth promotion in As-contaminated sites.

## 1. Introduction

Arsenic (As) is a ubiquitous toxic metalloid which is also released from several industrial processes such as mining, tanning of hides, and combustion of coal. Due to these reasons, the level of As in drinking waters of many areas has been increased beyond WHO standard drinking water limits (0.01 mg L^−1^) [1]. As is primarily found in two major forms: arsenate [As(V)] and arsenite [As(III)] [2]. As(III) is reported to be ~100 times more toxic as compared to As(V) [3, 4]. As(V) causes harm to the cells by being analogous to phosphorous, replacing the latter in many chemical reactions [5]. As(III), on the other hand, affects activity of many enzymes by reacting with their sulfhydryl groups [6]. As is toxic to all life forms, including plants, animals, and human beings. Long-term exposure to As, especially through drinking water, can cause arsenicosis which is manifested as different types of cancers and skin allergies. In plants, the toxicity of As causes reduced growth and low yields [7]. As is toxic to microorganisms as well; however, several bacteria are known to resist and detoxify As. As-resistant bacteria are known to have effects on As-oxidation/reduction, methylation, and demethylation, as well as sorption and desorption as a result of their survival strategies against As-toxicity [8]. Such As-resistant bacteria are ideal for the bioremediation of As-contaminated lands. However, given the possible complexity and severity of the As-polluted sites, the bacteria should be metabolically diverse in order to thrive in different environments.

Purple nonsulfur bacteria (PNSB) are one of the most metabolically diverse and adaptable bacteria. They are phototrophic and are widely found in nature, especially in submerged habitats that are exposed to sunlight, such as rice paddies, sewage dumping sites, and riverbeds. These bacteria have an extensive range of growth modes and are capable of growing photoautotrophically/photoheterotrophically under anaerobic conditions in presence of incandescent light utilizing a variety of organic substrates and can also grow chemoorganotrophically under aerobic dark conditions [9]. These properties make them ideal for wastewater bioremediation [10]. Several strains of PNSB are also known to have plant growth promoting abilities in terms of nitrogen fixation [11], phosphate solubilization, and production of phytohormones such as indole-3-acetic acid and 5-aminolevulinic (ALA) [12]. The most common genera are* Rhodobacter* and* Rhodopseudomonas*, detected in 73% and 80% of the samples, respectively [13]. Some PNSB have also been reported to resist and detoxify several toxic compounds such as As [14]. Consequently, PNSB can also play a substantial role in As-biogeochemistry and bioremediation.

Isolation and characterization of metabolically diverse PNSB having abilities not only to detoxify As but also to support plant growth could be very useful for bioremediation purposes. This could help in reclamation of As-polluted sites for agriculture purposes.

## 2. Materials and Method

### 2.1. Sampling

Sampling was done from the fish pond near the University of the Punjab, Quaid-e-Azam campus, Lahore, Pakistan (31°29′55.4′′N 74°17′31.9′′E). Sampling location in the pond was so selected that it was shallow enough to allow penetration of sunlight and deep enough to have anoxic conditions, perfect for PNSB. Thus, 3-4 feet deep water and sediment samples were collected. Temperature and pH of the samples were recorded. As(V) and As(III) were purchased as Na_2_HAsO_4_·7H_2_O and NaAsO_2_, respectively. All the experiments were performed in triplicate.

### 2.2. Enrichment and Isolation of PNSB

Media suggested by Biebl and Pfennig [15] with slight modifications was used. The media contained freshly prepared basal medium (0.33 gm KH_2_PO_4_, 0.33 gm MgSO_4_·7H_2_O, 0.33 gm NaCl, 0.05 gm CaCl_2_·2H_2_O, 0.5 gm NH_4_Cl, 1.0 gm sodium succinate, and 0.02 gm yeast extract L^−1^) to which 0.5 mL FeSO_4_·7H_2_O (0.02%) and 1.0 mL trace salt solution (10.0 mg ZnSO_4_·7H_2_O, 3.0 mg MnSO_4_·4H_2_O, 30.0 mg H_3_BO_3_, 20.0 mg CoCl_2_·6H_2_O, 2.0 mg NiCl_2_·6H_2_O, 3.0 mg Na_2_MoO_4_ L^−1^) were added and autoclaved. After autoclaving, while still warm, 450 mL of media was aseptically poured in autoclaved 500 mL Schott bottles. Media were allowed to cool and 50 mL of each sample (10% inoculum, water plus sediment) was inoculated aseptically, leaving little headspace. Media were overlaid with mineral oil and incubated at 28°C in the presence of incandescent light. Once the enrichment, reddish maroon colored bloom, was achieved (~10 days incubation), samples were drawn aseptically, serially diluted, and plated on Pfennig agar media plates. Media supplemented with different carbon sources (sodium acetate, sodium citrate, maleic acid, sodium succinate, and sodium lactate) were also used to conclude which carbon source is more suitable for the growth of PNSB. Plates were incubated at 28°C for 5–7 days in anaerobic jars (Oxoid) containing anaerobic sachet (Merck) in incandescent light. After incubation, PNSB were purified by streak plate method and characterized. PNSB were then cultured on Pfennig agar media (succinate) containing 1.0 mM As(V). PNSB that were able to grow in presence of As(V) were selected for further studies.

### 2.3. Minimum Inhibitory Concentration (MIC) of As(V)

Pfennig media containing 50 mM, 100 mM, or 150 mM As(V) were prepared and dispensed in screw capped tubes followed by autoclaving. The media were inoculated with overnight cultures, overlaid with mineral oil, and incubated at 28°C in incandescent light for 6-7 days. Presence or absence of growth was recorded.

### 2.4. As-Oxidation/Reduction Assay

#### 2.4.1. Qualitative Assay

Mineral medium [16], supplemented with 10 mM As(V) or 5 mM As(III), was inoculated with PNSB and incubated at 37°C for 48 hours. After incubation, 200 *μ*L of each supernatant was taken in microtiter plate wells and mixed with 0.01 M 12 *μ*L KMNO_4_. Color development was observed and recorded. A change from purple to yellowish brown color indicates As(V) reduction to As(III) [17].

#### 2.4.2. Quantitative Assay

Overnight PNSB cultures were harvested and pellets were washed (twice) with and resuspended in normal saline. Cell suspensions were divided into two sets of tubes: one set was supplemented with 10.0 mM As(V) and the other set was supplemented with 5.0 mM As(III) followed by incubation at 37°C for 5 hours. Samples were drawn at the start and after incubation. Samples were centrifuged and supernatants were analyzed for estimation of As(V) oxidation/reduction as reported by Cummings et al. [18] with slight modifications. Standard curve was created using known concentrations of As(V) ranging from zero to 1000 mM As(V). 100 *μ*L of sample was mixed with 100 *μ*L of 24 mM HCl followed by immediate vortexing. 100 *μ*L of the acidified sample was mixed with 900 *μ*L of the coloring reagent followed by immediate vortexing. The samples were incubated at 78°C in a water bath for 20 minutes and kept on ice for 5 minutes. Samples were quantified at 865 nm using spectrophotometer (CECIL CE 7200).

### 2.5. Determination of Cross Metal Resistance

Selected isolates were tested for resistance against other metals. Media containing 1.0 mM Co(NO_3_)_2_·6H_2_O, ZnSO_4_·7H_2_O, NiCl_2_·6H_2_O, CdCl_2_·5H_2_O, K_2_CrO_4_, or CuSO_4_·5H_2_O each were prepared. The media flasks were inoculated with overnight broth cultures and incubated at 37°C for 72 hours. After completion of incubation, absence or presence of growth was recorded.

### 2.6. 16S rRNA Gene Sequencing and Phylogenetic Analysis

16S rRNA gene of selected PNSB was sequenced from Macrogen (Korea) using primers 518F (CCAGCAGCCGCGGTAATACG) and 800R (TACCAGGGTATCTAATCC). Base call quality was checked though FinchTV. Sequences were classified using NCBI BLAST (https://blast.ncbi.nlm.nih.gov/Blast.cgi?PAGE_TYPE=BlastSearch) and nearest homologues were downloaded. Multiple sequence alignment was done in ClustalW. Neighbour-Joining (NJ) phylogenetic tree [19] was made in MEGA 5.0 [20] with 100 bootstrap replicates [21] as an estimate of branch support.

### 2.7. Plant Growth Promoting Attributes of PNSB

#### 2.7.1. Auxin Production

LB-broth supplemented with 500 mg mL^−1^ tryptophan was prepared and inoculated with overnight PNSB cultures followed by 7 days of incubation at 37°C on 150 rpm agitation. Following incubation, cultures were centrifuged and 100 *μ*L supernatant was mixed with 200 *μ*L Salkowski's reagent (1.0 mL 0.05 M FeCl_3_, 50 mL 35% HClO_4_) [22] in microtiter plate followed by incubation in dark for 30 minutes. Appearance of pinkish color indicated auxin production. Absorbance was measured at 535 nm using Epoch BioTek plate reader.

#### 2.7.2. Phosphate Solubilization

PNSB were streaked on Pikovskaya [23] media followed by incubation at 28°C for 7 days. Following incubation, plates were observed for the presence or absence of clear zones around PNSB colonies, an indication of phosphate solubilization.

#### 2.7.3. Hydrogen Cyanide (HCN) Production

PNSB were swabbed on nutrient agar supplemented with glycine (4.4 gm L^−1^) and a filter paper soaked in 2.0% Na_2_CO_3_ solution (in 0.5% picric acid) was placed on the top of the agar surface [24, 25]. After incubation at 28°C for 4 days, development of orange to red color of the filter paper indicated the positive result for HCN production.

### 2.8. Plant-Microbe Interaction Experiments

PNSB were cultured in Pfennig broth media as described earlier. Cells were harvested and resuspended in autoclave water to achieve 0.5_600 nm_ optical density.* Vigna mungo* seeds, purchased from Punjab Seed Corporation, were surface-sterilized with 0.1% HgCl_2_ for 2.0 minutes with continuous shaking followed by washing with distilled water, 3-4 times. Seeds were incubated in separate as well as in mixed PNSB suspensions (consortium) for 15–20 minutes at room temperature. Following incubation, seeds were sown in 16 cm wide and 13.5 cm deep pots each containing equal amount of sieved soil. Eight seeds were sown per pot and after germination plants were thinned to five plants per pot. Pots were divided into 4 sets: (a) pots containing uninoculated seeds, (b) pots containing inoculated seeds, (c) pots containing uninoculated seeds and As [1.0 mM As(V) and 0.1 mM As(III)], and (d) the pots containing inoculated seeds as well as As [1.0 mM As(V) and 0.1 mM As(III)]. Each set contained 10 pots. The pots were placed in 12-hour photoperiod at 25 ± 2°C for two weeks. Watering was done regularly.

### 2.9. Statistical Analysis

Results were analyzed for statistical significance using analysis of variance (ANOVA), followed by post hoc analysis using two-tailed *t*-tests (*p* = 0.05) in order to compare different treatments with each other. The threshold of significance was also adjusted using Bonferroni correction. All the biostatistical analysis was done using Microsoft Excel 2013 data analysis toolkit.

## 3. Results

### 3.1. Isolation and Identification of As-Resistant PNSB

Purplish maroon colored bloom was achieved in the enrichment Schott bottles after incubation of 10 days at 28°C in incandescent light. Media containing sodium acetate and sodium lactate did not show any growth. Malic acid containing media gave ~10.8 × 10^6^ CFU mL^−1^ and sodium succinate containing media gave ~7 × 10^7^ CFU mL^−1^, whereas CFU mL^−1^ on sodium citrate containing media was 2 × 10^7^ (Table 1). All the isolated PNSB were catalase and oxidase positive. All isolates were Gram-negative and nonspore former (Table 1).

**Figure 1:**
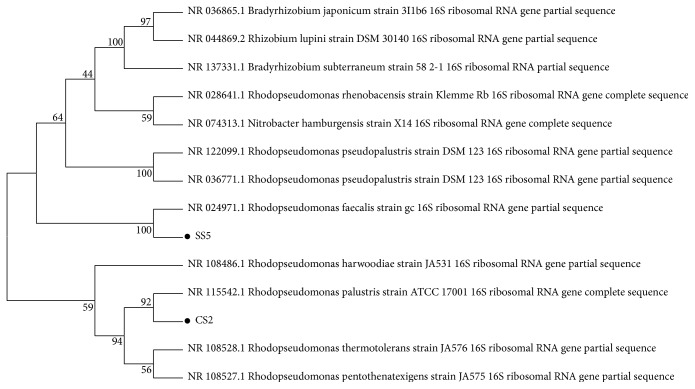
NJ-phylogenetic tree of PNSB isolates CS2 and SS5 along with their closest homologues from NCBI nucleotide BLAST results. Sequences were aligned with ClustalW and tree was made in MEGA 5 using 100 bootstrap replicates. Isolate CS2 grouped with* Rhodopseudomonas palustris* and SS5 grouped with* Rhodopseudomonas faecalis.*

16S rRNA sequence analysis of the PNSB showed that isolate CS2 showed maximum homology to* Rhodopseudomonas palustris* (99%), whereas isolate SS5 showed maximum homology to* Rhodopseudomonas faecalis* (99%), when aligned through nucleotide BLAST tool of NCBI. The results were confirmed by making NJ-phylogenetic tree. Isolate CS2 grouped with* R. palustris*, whereas isolate SS5 grouped with* R. faecalis* (Figure 1). The sequences were submitted in NCBI GenBank under the accession numbers KY098792 and KY098793 for* R. palustris* CS2 and* R. faecalis* SS5, respectively.

### 3.2. Heavy Metal Resistance

**Figure 2:**
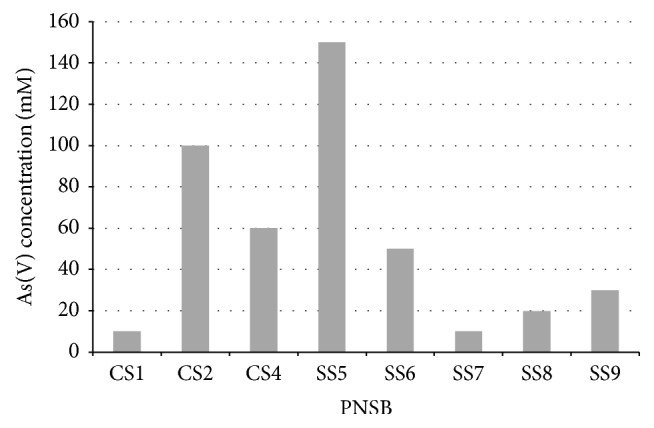
Minimum inhibitory concentration of As(V) against PNSB. Highest concentration of As(V) was resisted by* R. faecalis* SS5 and* R. palustris* CS2, up to 150 and 100 mM, respectively.

Maximum As(V) resistance was shown by* R. faecalis* SS5, up to 150 mM, whereas* R. palustris* CS2 resisted up to 100 mM As(V) (Figure 2). All the isolates were resistant to 1.0 mM Cr, Ni, and Zn, while they were sensitive to the Cu, Cd, and Co.

### 3.3. Estimation of As-Oxidation/Reduction

KMNO_4_ was used as reagent which changes its color to yellow in presence of As(III) but retains its color in the presence of As(V).* R. palustris* CS2 was found to be As(V) reducers, while* R. faecalis* SS5 was found to be As(III)-oxidizer (Table 1).

**Figure 3:**
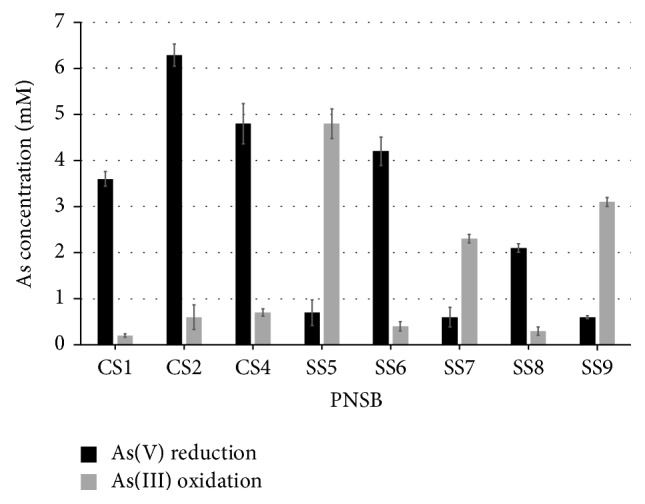
As-oxidation/reduction by PNSB. Bacterial cell suspensions were incubated for 5 hours in normal saline containing either 10 mM As(V) or 5 mM As(III). Highest As(V) reduction was shown by* R. palustris* CS2 up to 62.9% (6.29 ± 0.24 mM), whereas highest As(III) oxidation was shown by* R. faecalis* SS5 up to 96% (4.8 ± 0.32 mM).

Isolates* R. palustris* CS2 showed highest As(V) reduction potential and reduced 62.9% (6.29 ± 0.24 mM) As(V) in 5 hours of incubation. Isolates CS4 and SS6 reduced 48% (4.8 ± 0.4 mM) As(V), whereas isolate SS6 reduced 42% (4.2 ± 0.31 mM) As(V). Maximum As(III) oxidation was exhibited by* R. faecalis* SS5 and oxidized 96% (4.8 ± 0.32 mM) As(III) in 5 hours of incubation (Figure 3).

### 3.4. Plant Growth Promoting Activities of PNSB

**Figure 4:**
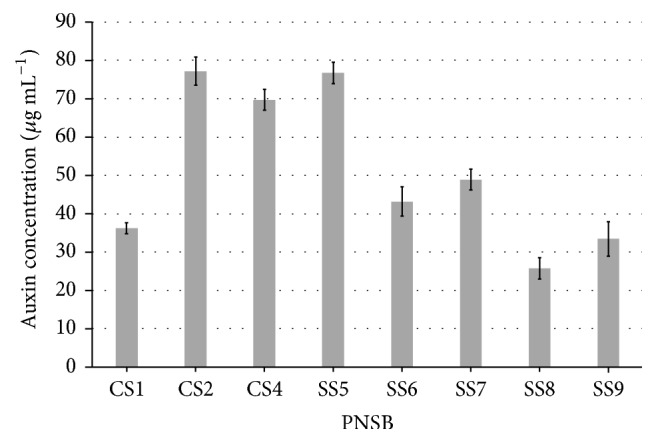
Auxin production by As-resistant PNSB.* R. palustris* CS2 and* R. faecalis* SS5 produced highest auxin concentrations up to 77.18 ± 3.7 and 76.67 ± 2.8 *μ*g mL^−1^.

Isolate* R. faecalis* SS5 was strongly positive for the HCN production, while* R. palustris* CS2 was weakly positive as indicated by the intensity of the orange color developed on the filter paper (Table 1). All the isolates were positive for auxin production as well and maximum auxin production was exhibited by* R. palustris* CS2 (77.18 ± 3.7 *μ*g mL^−1^) and* R. faecalis* SS5 (76.67  ±  2.8 *μ*g mL^−1^) (Figure 4). All the isolates were negative for phosphate solubilization (Table 1).

### 3.5. Effects of PNSB on Plant Growth

Different sets of pots showed different germination rates according to the conditions provided. Germination rate was affected in the presence of As (data not shown).

**Figure 5:**
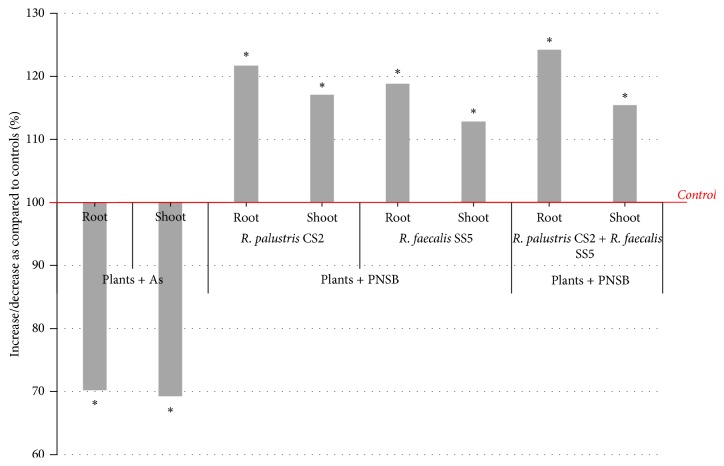
Effects of As and PNSB on* Vigna mungo* growth as compared to the control plants (control = 100%, plants exposed neither to As nor PNSB). In presence of As, shoot and root lengths decreased up to 30.7% and 29.7%, respectively, as compared to the control plants. In presence of* R. palustris* CS2, shoot and root lengths increased up to 17 and 21.7%, whereas in presence of* R. faecalis* SS5 shoot and root lengths increased up to 12.8 and 18.8%, respectively. In presence of both* R. palustris* CS2 and* R. faecalis* SS5, shoot length and root length increased up to 15.4 and 24.2%, respectively. ^*∗*^Significantly different from control, *p* < 0.05.

Control plants were exposed neither to As nor to PNSB. Average shoot length was 24.0 ± 0.9 cm, while average root length was 5.8  ±  0.5 cm. Increase in shoot and root length was observed in the presence of PNSB.* R. palustris* CS2 treated plants showed 17% (28.1 ± 0.87 cm) increase in shoot length and 21.7% (7.07  ±  0.42 cm) increase in root length as compared to the control plants.* R. faecalis* SS5 treated plants showed 12.8% (27.09 ± 0.81 cm) increase in shoot length and 18.8% (6.9 ± 0.34 cm) increase in root length as compared to the control plants. Shoot length of the plants treated with mixed isolates (*R. palustris* CS2 and* R. faecalis* SS5) increased up to 15.4% (27.7 ± 0.67 cm), whereas root length increased up to 24.2% (7.2 ± 0.52 cm) as compared to the control plants (Figure 5).

**Figure 6:**
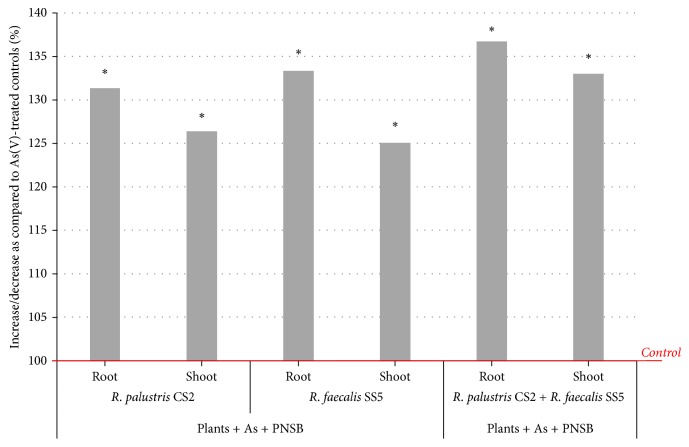
Effects of As-resistant PNSB on* Vigna mungo* growth in presence of As compared to the control plants (control = 100%, plants exposed to As).* R. palustris* CS2 treated plants showed 26.3 and 31.3% increase in shoot and root length, respectively, whereas* R. faecalis* SS5 treated plants showed 25 and 33.3% increase in shoot and root length, respectively, as compared to the control plants. Shoot and root lengths of plant treated with both* R. palustris* CS2 and* R. faecalis* SS5 increased up to 33% and 36.7%, respectively. ^*∗*^Significantly different from control, *p* < 0.05.

Shoot and root lengths of plants exposed to As decreased up to 30.7% (16.6 ± 0.58 cm) and 29.7% (4.08 ± 0.76 cm), respectively. Plants exposed to both As(V) and PNSB showed increased root and shoot lengths. Shoot length of plants treated with As and* R. palustris* CS2 increased up to 26.3% (21.0 ± 1.1 cm), while root length increased up to 31.3% (5.3 ± 0.4 cm) as compared to the control plants (plants exposed to As).* R. faecalis* SS5 inoculated plants showed 25% (20.7 ± 1.4 cm) increase in shoot length and 33.3% (5.4 ± 0.65 cm) increase in root length in presence of As as compared to control plants. Shoot and root lengths of plants treated with mixed isolates in presence of As increased up to 33% (22.1 ± 0.7 cm) and 36.7% (5.5 ± 0.4 cm), respectively (Figure 6).

ANOVA of all the plant sets was significant, and Bonferroni corrected posttest *t*-test indicated that the length of plants exposed to As was significantly lower and the length of plants exposed to PNSB was significantly higher, compared to control plants (not exposed to As) (*p* ≤ 0.05). The statistical analysis also confirmed that the length of plants exposed to both As and PNSB was significantly higher as compared to plants exposed to As only (*p* ≤ 0.05).

Wet and dry weight of plants exposed to neither As nor PNSB was 1.4 ± 0.1 gm and 0.2 ± 0.05 gm, respectively.* R. palustris* CS2 inoculated plants had 1.7 ± 0.1 gm wet weight, while the dry weight was 0.3 ± 0.03 gm. Wet weight of* R. faecalis* SS5 treated plants was 1.6 ± 0.2 gm, whereas the dry weight was 0.2 ± 0.04 gm.

Wet and dry weight of plants exposed to As was 0.9 ± 0.08 gm and 0.09 ± 0.01 gm, respectively.* R. palustris* CS2 treated plants had 1.25 ± 0.8 gm wet weight in the presence of As, while the dry weight was 0.14 ± 0.03 gm. Wet weight of* R. faecalis* SS5 treated plants, in presence of As, was 1.1 ± 0.3 gm, whereas the dry weight was 0.12 ± 0.07 gm.

## 4. Discussion

Resistance against As(V) and plant growth promoting activities, such as auxin and HCN production, were the main parameters used for the screening and selection of PNSB. PNSB are known to utilize different carbon sources including those of TCA cycle, as well as different fatty acids and even fix CO_2_ for their growth [26, 27]. In the present study, As-resistant PNSB isolates were able to utilize malic acid, sodium succinate, or sodium citrate. The isolates showing best results were identified as* R. palustris* CS2 and* R. faecalis* SS5 by aligning their 16S rRNA gene sequences with the NCBI nucleotide database using BLAST tool.* Rhodopseudomonas* and many other PNSB belong to*α-proteobacteria*; however, some PNSB are also found in*β-proteobacteria* [28]. Both qualitative and quantitative assays of As-oxidation/reduction showed that* R. palustris* CS2 had ability to reduce As(V) into As(III), whereas* R. faecalis* SS5 had the potential to oxidize As(III) to As(V). Genetic determinants of As-resistance and detoxification are found in the form of an operon called* ars* operon; thus the ability to resist and detoxify As is mostly linked together [29]. Bacteria are known to oxidize as well as reduce As, and even some bacteria are reported to perform both functions [30, 31]. Thus PNSB have important roles to play in the biogeochemical cycling of As as well.

Bacteria are also known to produce different phytohormones that enhance plants' growth [32]. IAA, a type of auxin, exhibits one of the greatest plant growth promoting activity [33]. Both isolates* R. palustris* CS2 and* R. faecalis* SS5 showed highest level of auxin production. Hydrogen cyanide is another secondary metabolite important for plant growth.* R. palustris* CS2 and* R. faecalis* SS5 were also found to produce hydrogen cyanide. Plant growth promoting ability of these isolates was checked with* V. mungo* due to its fast growth rate. Both* R. palustris* CS2 and* R. faecalis* SS5 were able to enhance the growth of plants both in presence and in absence of As. The increase in the growth of plants due to PNSB in presence of As could be due to the reason that PNSB were able to oxidize/reduce As present in the soil, thus decreasing the bioavailability of As to the plants. Moreover, as these PNSB were also capable of producing phytohormones, this added extra benefit for the growth of the plants. Combined effect of* R. palustris* CS2 and* R. faecalis* SS5 was more pronounced for root enhancement. This could be due to the fact that mixed bacteria were able to oxidize As(III) as well as reduce As(V), keeping a redox cycle and thus further decreasing the bioavailability of As to the plants. Table S1 (see Supplementary Material available online at https://doi.org/10.1155/2017/6250327) shows some of the studies that report As-oxidation/reduction or plant growth promotion by PNSB. As the table indicates, many* Rhodopseudomonas* and* Rhodobacter* species are known to support plant growth by producing phytohormones. Nookongbut et al. [14] reported As-resistance and detoxification by different strains of* R. palustris*. Wong et al. [34] reported IAA production as well as plant growth enhancement by* R. palustris*. However, literature reporting both As-detoxification and plant growth promotion by same PNSB strains is scarce. The present study reports this important aspect of PNSB strains which can be utilized to enhance plant growth in As-contaminated areas.

## 5. Conclusions

Metabolically diverse bacteria such as PNSB with ability to not only detoxify As but also support plant growth could be useful both in bioremediation of As-contaminated lands and in supporting plant growth in such contaminated sites.

## Supplementary Material

Table S1 shows some of the studies that reported either As-oxidation/reduction or plant growth promotion by PNSB. As the table indicates, many Rhodopseudomonas and Rhodobacter species are known to support plant growth by producing phytohormones. However, literature reporting both As-detoxification and plant growth promotion by same PNSB strains is scarce.

## Figures and Tables

**Table 1:** Characteristics of As-resistant PNSB isolated in this study.

Strain	Gram reaction	Spore staining	Catalase	Oxidase	Auxin production	HCN production	Phosphate	As(V)-reduction	As(III)-oxidation
CS1	Gram-negative	−	Positive	Positive	+	+++	−	+++	−
CS2	Gram-negative	−	Positive	Positive	+	++	−	+++	−
CS4	Gram-negative	−	Positive	Positive	+	−	−	+++	−
SS5	Gram-negative	−	Positive	Positive	+	+++	−	−	+++
SS6	Gram-negative	−	Positive	Positive	+	−	+	+++	−
SS7	Gram negative	−	Positive	Positive	+	++	−	−	+
SS8	Gram-negative	−	Positive	Positive	+	−	−	+	−
SS9	Gram-negative	−	Positive	Positive	+	−	−	−	+
