# Supplementary material for: Arsenic-Redox Transformation and Plant Growth Promotion by Purple Nonsulfur Bacteria Rhodopseudomonas palustris CS2 and Rhodopseudomonas faecalis SS5
Source: Biomed Res Int. 2017 Mar 12;2017:6250327. doi: 10.1155/2017/6250327 (PMC5366193; doi:10.1155/2017/6250327)
Supplement: Supplementary file 1 — Table S1 shows some of the studies that reported either As-oxidation/reduction or plant growth promotion by PNSB. As the table indicates, many Rhodopseudomonas and Rhodobacter species are known to support plant growth by producing phytohormones. However, literature reporting both As-detoxification and plant growth promotion by same PNSB strains is scarce. [file 6250327.f1.docx]

**Table S1. Some of the reported As-resistant and/or plant growth promoting PNSB**

| **Author** | **PNSB** | **As(V) resistance** | **As(III)**  **Resistance** | **As(V) reduction** | **As(III) oxidation** | **Auxin production** | **Phosphate solublization** | **HCN production** | **Plant growth promotion** |
| --- | --- | --- | --- | --- | --- | --- | --- | --- | --- |
| [Nookongbut, et al. [1](#_ENREF_1)] | *Rhodopseudomonas palustris* | **Dark conditions:** As(V)-MIC 20,000 mg L^-1^),  **Light conditions:**  As(V)-MIC 2,000 mg L^-1^). | **Dark conditions:**  As(III)-MIC 2,500 mg L^-1^  **Light conditions:** As(III)-MIC: 350 mg L^-1^ | 93.58% (200mg L^-1^) | 53.76% (100mg L^-1^) | NA | NA | NA | NA |
| [Lee, et al. [2](#_ENREF_2)] | *R. palustris* | NA | NA | NA | NA | NA | NA | NA | 10–27 and 22–40% increase of fresh and dry weights of shoots, respectively |
| [Quambusch, et al. [3](#_ENREF_3)] | *R. palustris* | NA | NA | NA | NA | NA | NA | NA | 6.4 (±2.8)  roots per shoot compared to 4.4 (±1.9) roots in control shoots |
| [Xu, et al. [4](#_ENREF_4)] | *R. palustris* | NA | NA | NA | NA | ALA: + | NA | NA | + |
| [Nunkaew, et al. [5](#_ENREF_5)] | *R. palustris* | NA | NA | NA | NA | ALA: 25.67µM | NA | NA | NA |
| [Zhao, et al. [6](#_ENREF_6)] | *R. palustris* | 2.44mM | 1.55mM | NA | NA | NA | NA | NA | NA |
| [Wong, et al. [7](#_ENREF_7)] | *R. palustris* | NA | NA | NA | NA | IAA: 208.4 ± 41.3µM | NA | NA | 50% increase in shoot length |
| [Lin, et al. [8](#_ENREF_8)] | *R. capsulatus* | 2.03Mm | 0.61mM | + | NA | NA | NA | NA | NA |
| [Xu, et al. [9](#_ENREF_9)] | *R. palustris* | NA | NA | NA | NA | IAA: 37-64.4mg L^-1^ | NA | NA | NA |
| [Kantha, et al. [10](#_ENREF_10)] | *R. palustris* | NA | NA | NA | NA | ALA: + | NA | NA | NA |
| [Choorit, et al. [11](#_ENREF_11)] | *R. palustris* | NA | NA | NA | NA | **Dark condition:** ALA: 89.81μM molC^-1^  **Light condition:** ALA: 245.75μM molC^-1^ | NA | NA | NA |
| [Kantha, et al. [12](#_ENREF_12)] | *R. palustris* | NA | NA | NA | NA | ALA: 2.96mM | NA | NA | + |
| [Saikeur, et al. [13](#_ENREF_13)] | *R. palustris* | NA | NA | NA | NA | ALA: 182.91µM | NA | NA | NA |
| [Lee, et al. [14](#_ENREF_14)] | *Rhodopseudomonas* sp. | NA | NA | NA | NA | NA | + | NA | 78.6 and 120.6% increase in dry and wet weights,  34.6% increase in shoot length |
| [Harada, et al. [15](#_ENREF_15)] | *R. palustris* | NA | NA | NA | NA | NA | NA | NA | + |

NA = Data not available; + = Positive; - = Negative; IAA: Indole acetic acid; ALA = 5-aminolevulinic

REFERENCES

1. P. Nookongbut, D. Kantachote and M. Megharaj, "Arsenic contamination in areas surrounding mines and selection of potential As-resistant purple nonsulfur bacteria for use in bioremediation based on their detoxification mechanisms," *Annals of Microbiology*, pp. 1-11, 2016.

2. S.-K. Lee, H.-S. Lur, K.-J. Lo, K.-C. Cheng, C.-C. Chuang, S.-J. Tang, Z.-W. Yang and C.-T. Liu, "Evaluation of the effects of different liquid inoculant formulations on the survival and plant-growth-promoting efficiency of *Rhodopseudomonas palustris* strain PS3," *Applied microbiology and biotechnology*, pp. 1-11, 2016.

3. M. Quambusch, J. Brümmer, K. Haller, T. Winkelmann and M. Bartsch, "Dynamics of endophytic bacteria in plant in vitro culture: quantification of three bacterial strains in *Prunus avium* in different plant organs and in vitro culture phases," *Plant Cell, Tissue and Organ Culture (PCTOC)*, pp. 1-13, 2016.

4. J. Xu, Y. Feng, Y. Wang, X. Luo, J. Tang and X. Lin, "The foliar spray of *Rhodopseudomonas palustris* grown under *Stevia* residue extract promotes plant growth via changing soil microbial community," *Journal of Soils and Sediments*, vol. 16, no. 3, pp. 916-923, 2016.

5. T. Nunkaew, D. Kantachote, T. Nitoda and H. Kanzaki, "Selection of salt tolerant purple nonsulfur bacteria producing 5-aminolevulinic acid (ALA) and reducing methane emissions from microbial rice straw degradation," *Applied Soil Ecology*, vol. 86, pp. 113-120, 2015.

6. C. Zhao, Y. Zhang, Z. Chan, S. Chen and S. Yang, "Insights into arsenic multi-operons expression and resistance mechanisms in *Rhodopseudomonas palustris* CGA009," *Frontiers in Microbiology*, vol. 6, 2015.

7. W.-T. Wong, C.-H. Tseng, S.-H. Hsu, H.-S. Lur, C.-W. Mo, C.-N. Huang, S.-C. Hsu, K.-T. Lee and C.-T. Liu, "Promoting effects of a single *Rhodopseudomonas palustris* inoculant on plant growth by *Brassica rapa chinensis* under low fertilizer input," *Microbes and Environments*, vol. 29, no. 3, pp. 303, 2014.

8. H. Z. Lin, Y. H. Yue, J. C. Lü, G. C. Zhao and P. S. Yang, "Variation in composition and relative content of accumulated photopigments in a newly isolated *Rhodobacter capsulatus* strain XJ-1 in response to arsenic," *Journal of Environmental Science and Health, Part A*, vol. 49, no. 13, pp. 1493-1500, 2014.

9. J. Xu, Y. Feng, Y. Wang and X. Lin, "Characteristics of purple nonsulfur bacteria grown under *Stevia* residue extractions," *Letters in applied microbiology*, vol. 57, no. 5, pp. 420-426, 2013.

10. T. Kantha, C. Chaiyasut, D. K. S. Sukrong and A. Muangprom, "Synergistic growth of lactic acid bacteria and photosynthetic bacteria for possible use as a bio-fertilizer," *African Journal of Microbiology Research*, vol. 6, no. 3, pp. 504-511, 2012.

11. W. Choorit, A. Saikeur, P. Chodok, P. Prasertsan and D. Kantachote, "Production of biomass and extracellular 5-aminolevulinic acid by *Rhodopseudomonas palustris* KG31 under light and dark conditions using volatile fatty acid," *Journal of bioscience and bioengineering*, vol. 111, no. 6, pp. 658-664, 2011.

12. T. Kantha, C. Chaiyasut, D. Kantachote, S. Sukrong and A. Muangprom, "Selection of photosynthetic bacteria producing 5-aminolevulinic acid from soil of organic saline paddy fields from the Northeast region of Thailand," *African Journal of Microbiology Research*, vol. 4, no. 17, pp. 1848-1855, 2010.

13. A. Saikeur, W. Choorit, P. Prasertsan, D. Kantachote and K. Sasaki, "Influence of precursors and inhibitor on the production of extracellular 5-aminolevulinic acid and biomass by *Rhodopseudomonas palustris* KG31," *Bioscience, biotechnology, and biochemistry*, vol. 73, no. 5, pp. 987-992, 2009.

14. K.-H. Lee, R.-H. Koh and H.-G. Song, "Enhancement of growth and yield of tomato by *Rhodopseudomonas* sp. under greenhouse conditions," *The Journal of Microbiology*, vol. 46, no. 6, pp. 641-646, 2008.

15. N. Harada, M. Nishiyama, S. Otsuka and S. Matsumoto, "Effects of inoculation of phototrophic purple bacteria on grain yield of rice and nitrogenase activity of paddy soil in a pot experiment," *Soil Science and Plant Nutrition*, vol. 51, no. 3, pp. 361-367, 2005.
